# Model-Based Biomarker Selection for Dose Individualization of Tyrosine-Kinase Inhibitors

**DOI:** 10.3389/fphar.2020.00316

**Published:** 2020-03-12

**Authors:** Maddalena Centanni, Lena E. Friberg

**Affiliations:** Department of Pharmaceutical Biosciences, Uppsala University, Uppsala, Sweden

**Keywords:** tyrosine kinase inhibitor, dose individualization, population modeling, PKPD, pharmacometrics

## Abstract

Tyrosine-kinase inhibitors (TKIs) demonstrate high inter-individual variability with respect to safety and efficacy and would therefore benefit from dose or schedule adjustments. This study investigated the efficacy, safety, and economical aspects of alternative dosing options for sunitinib in gastro-intestinal stromal tumors (GIST) and axitinib in metastatic renal cell carcinoma (mRCC). Dose individualization based on drug concentration, adverse effects, and sVEGFR-3 was explored using a modeling framework connecting pharmacokinetic and pharmacodynamic models, as well as overall survival. Model-based simulations were performed to investigate four different scenarios: (I) the predicted value of high-dose pulsatile schedules to improve clinical outcomes as compared to regular daily dosing, (II) the potential of biomarkers for dose individualizations, such as drug concentrations, toxicity measurements, and the biomarker sVEGFR-3, (III) the cost-effectiveness of biomarker-guided dose-individualizations, and (IV) model-based dosing approaches versus standard sample-based methods to guide dose adjustments in clinical practice. Simulations from the axitinib and sunitinib frameworks suggest that weekly or once every two weeks high-dosing result in lower overall survival in patients with mRCC and GIST, compared to continuous daily dosing. Moreover, sVEGFR-3 appears a safe and cost-effective biomarker to guide dose adjustments and improve overall survival (€36 784.- per QALY). Model-based estimations were for biomarkers in general found to correctly predict dose adjustments similar to or more accurately than single clinical measurements and might therefore guide dose adjustments. A simulation framework represents a rapid and resource saving method to explore various propositions for dose and schedule adjustments of TKIs, while accounting for complicating factors such as circulating biomarker dynamics and inter-or intra-individual variability.

## Introduction

Tyrosine-kinase inhibitors (TKIs) represent a group of targeted therapies that have shown great benefit in the treatment of various malignancies (Arora and Scholar, 2005). Contrary to most cytotoxic drugs, TKIs can be taken orally, and they target fundamental proteins involved in cancer cell proliferation, differentiation, migration, and metabolism. Despite their advantages, TKIs demonstrate high inter-individual variability in pharmacokinetics (PK) as well as in pharmacodynamics (PD), i.e. with respect to safety and efficacy outcomes, and, therefore, would benefit from dose individualization to optimize treatment outcomes (Sabanathan et al., 2017; Schmidinger et al., 2017).

Several proposals for dose individualization of TKIs have been made, based on PK and PD observations. Therapeutic drug monitoring/management (TDM) of drug plasma concentration appears an obvious choice given the large variability in drug exposure and the apparent exposure–response relationships (Verheijen et al., 2017). The drug exposure may be the area under the curve (AUC) or the pre-dose concentration at steady-state (C_trough_). TDM targets have indeed been suggested for TKIs in previous exposure–response studies. Dose individualization based on PD-biomarkers, such as soluble biomarkers or changes in blood pressure and neutrophil count, has also been proposed for several TKIs (Hansson et al., 2013a; Hansson et al., 2013b; Tsuchiya et al., 2015; Sabanathan et al., 2017; Schmidinger et al., 2017). Challenges in the evaluation of circulating biomarkers reside in (i) the time-related changes (i.e. kinetics) of biomarkers, (ii) their inter- and intra-individual variability and (iii) the limited patient and funding resources compared to the large number of drugs to be investigated (Buyse et al., 2010; Almufti et al., 2014).

In parallel, modification of dosing schedules has been explored for TKIs (i.e. continuous daily dosing versus 4 weeks on, 2 week off, Houk et al., 2008; Nozawa and Uemura, 2012). More recently, intermittent administration (i.e. weekly or biweekly) of high-dose TKIs have been proposed (Rovithi et al., 2014; Rovithi et al., 2016). This type of schedule results in higher peak plasma concentrations, and is proposed to give rise to enhanced efficacy, possibly due to a second mechanism of action. It is however not clear what impact this type of schedule has on various adverse effects (AEs) and overall survival (OS). Moreover, biomarker target values might not hold across drug schedules, such as continuous and intermittent, given the various concentration-time profiles different regimens can result in.

In addition to potential changes in clinical outcome, monetary aspects related to dose and schedule adjustments are important to take into consideration (Centanni et al., 2019). In the interest of managing healthcare costs, it has become acknowledged that alternative dosing regimens could result in lower prescribing costs, whilst maintaining equivalent efficacy (Ratain et al., 2019). Cost-effectiveness analysis represents a separate tool that highlights the role of each dose and schedule adjustment from both a clinical and economic perspective (Hughes and Walley, 2001). Typically, however, such analyses employ relatively empirical (i.e. Markov) models to reproduce transition probability between disease stages or between AE grades. Because of this feature, they may lack the flexibility required to draw inferences related to cost-effectiveness of various drug schedules and individualization approaches.

The question of the best treatment schedule and individualization options for TKIs remains, and is complex to sort out, given the different dynamics of each biomarker and AEs. As an illustrative example, diastolic blood pressure was found to have low accuracy in guiding dose adaptation of sunitinib in gastro-intestinal stromal tumors (GIST) (Centanni et al., 2018). Furthermore, there are several AEs variables that need to be taken into consideration as they may hamper dose increments. This in turn give rise to marginal treatment benefits that would require large patient populations to reach adequate power in prospective clinical trials (Goulooze et al., 2016; Centanni et al., 2018). Given the fact that there are over 25 TKIs available, and most are approved for several treatment indications, the number of combinations to be explored would be extensive and require a large amount of monetary and patient resources.

PK/PD modeling and simulation represents a means to analyze available information in order to quantify the added value and dynamics of each dose individualization rationale and dosing schedule in a rapid, ethical and low-cost manner (Almufti et al., 2014; Bender et al., 2015; Standing, 2017; Centanni et al., 2019). Model-based PK/PD frameworks can facilitate schedule selection in the presence of toxicity, disease, and survival models that can emulate a compromise between safety and efficacy under each treatment schedule and biomarker in a structured way (Jänne et al., 2016; Centanni et al., 2018). Moreover, mechanistic PK/PD frameworks possess a more biological character, which aside from potential inaccuracies related to the models, would provide more reliable inferences when analyzing the cost-effectiveness of alternative dosing strategies (van Hasselt et al., 2015). Previous model-based simulations have demonstrated that at the current suggested threshold values, the sunitinib dose in GIST can best be individualized by pharmacodynamic biomarkers, such as neutrophil count or soluble vascular-endothelial growth factor (VEGFR)-3, as opposed to blood pressure and drug concentrations (Centanni et al., 2018).

Given the long treatment period of TKIs such as sunitinib, and the potential impact of AEs on daily life (fatigue, hand-foot syndrome [HFS]), further research on sunitinib in GIST is of importance to optimize the biomarker threshold values for dose adaptation. In addition, the existing model-based framework of axitinib for metastatic renal cell carcinoma (mRCC) can be adapted for similar purposes (Schindler et al., 2017). The following work utilizes the existing sunitinib and axitinib frameworks to evaluate the efficacy, safety, and economical aspects of the abovementioned dosing options. Current clinical protocols and hospital expenses are integrated to emulate real-life scenarios and maximize the direct clinical applicability of the results. In the first section of the work, labeled recommended dosages of axitinib and sunitinib are compared to high-dose intermittent schedules for development of treatment-related AEs and OS. Secondly, biomarker-based dose adjustments are performed to evaluate potential differences in clinical outcomes and thirdly the predictive accuracy of model-based biomarker adjustments is compared to that of regular sample-based methods, wherein dose is directly adjusted according to the measured biomarker plasma levels. Finally, a cost-effectiveness analysis of sunitinib is performed for the available biomarker-based dose interventions.

## Methods

### Setting Up the Sunitinib and Axitinib Framework

**Figure 1:**
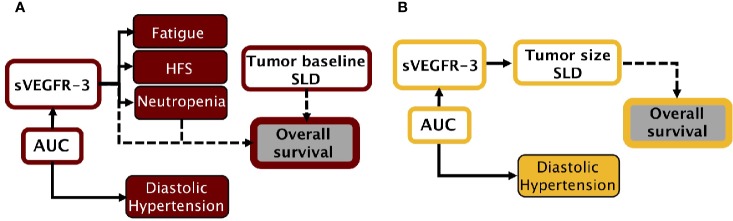
Simulation frameworks. **(A)** Model-based framework for sunitinib in GIST, adapted from previous work (Hansson et al., 2013a; Hansson et al., 2013b). **(B)** Model-based framework for axitinib in mRCC, adapted from previous work (Schindler et al., 2017). *AUC, area under the plasma drug concentration-time curve; dBP, diastolic blood pressure; HFS, hand-foot syndrome; SLD, sum of longest diameters (tumor); sVEGFR-3, soluble VEGFR-3*.

Two separate sunitinib simulation frameworks were built using a collection of previously created models (**Figure 1A**) (**Supplementary Material 1**) (Hansson et al., 2013a; Hansson et al., 2013b). None of the model parameters were re-estimated. Five AE models were available to describe the development of diastolic hypertension, neutropenia, thrombocytopenia, fatigue, and HFS under sunitinib therapy. Here, thrombocytopenia and diastolic hypertension were driven by sunitinib AUC, whereas remaining AEs were affected by changes in sVEGFR-3. A PK model of sunitinib and its active metabolite (SU12662) was selected to simulate changes in drug concentration over time (Yu et al., 2015). The time-courses of sVEGFR-3 and circulating levels of soluble KIT (sKIT) were described by two indirect response models. Longitudinal tumor size was reproduced with a tumor growth inhibition (TGI) model (Claret et al., 2009), affected by changes in sVEGFR-3, sKIT, and sunitinib AUC. Two OS models were divided over the simulation frameworks; one driven by changes in diastolic blood pressure (ΔdBP), scaled absolute change in neutrophil count (ANC), and baseline tumor size (Eqs. 1 and 2), and the second model was steered by relative changes in sVEGFR-3 (ΔsVEGFR-3) and baseline tumor size (Eq. 3). In the interest of clarity, dose adjustments based on the first (toxicity-related) framework will be referred to as toxicity-adjusted dosing (TAD), whereas dose-adjustments based on the second framework (sVEGFR-3-based) will be referred to as biomarker-based dosing (BAD). Lastly, a dropout model was added to both frameworks to account for the tendency of individuals with poorer responses to discontinue treatment. The probability to drop out was determined by tumor progression and tumor size.

(1) $$\text{ANC}(\text{t})=\frac{\text{ANC}(t)-5}{5}$$

(2) $$\Delta \text{dBP}(\text{t})=\frac{\text{dBP}(t)-{\text{BASE}}_{dBP}}{{\text{BASE}}_{dBP}}$$

(3) $$\Delta \text{sVEGFR-3(t)=}\frac{\text{sVEGFR-3(}t{\text{)-BASE}}_{sVEGFR-3}}{{\text{BASE}}_{sVEGFR-3}}$$

In a similar manner, the axitinib simulation framework was built on a collection of previous models, using originally estimated parameters (**Figure 1B**) (**Supplementary Material 2**) (Schindler et al., 2017). An axitinib PK model was included to simulate drug serum concentration over time (Garrett et al., 2014). We assumed axitinib was taken orally in the XLI formulation, which is the marketed formulation according to the label information. One AE model was available to describe the development of diastolic blood pressure under treatment. Changes in sVEGFR-3 were captured by an indirect response model. Tumor size (sum of longest diameters, SLD) was described by a growth model, affected by changes in sVEGFR-3. OS, driven by tumor size and progression, was simulated by means of a time-to-event model. Lastly, a dropout model, driven by axitinib exposure, tumor progression, and tumor size, was added to simulate patients who discontinued treatment.

Both the sunitinib and axitinib frameworks were implemented in the R-based package *mrgsolve* (version 0.8.10, Elmokadem et al., 2019), together with the parameter estimates. Visual audits were performed to compare the *mrgsolve* to NONMEM (Beal et al., 1989) simulation output to assure accurate translation of the models.

### Virtual Populations

In order to achieve the simulations, datasets containing 1,000 virtual individuals with metastatic and/or unresectable GIST, or mRCC, were generated using the dmutate (version 0.1.2) and dplyr (version 0.7.4) R packages. Individual covariates were created by sampling from a defined population distribution (Hansson et al., 2013a; Hansson et al., 2013b; Garrett et al., 2014; Yu et al., 2015; Schindler et al., 2017). Weight was assumed to follow a normal distribution with a mean of 73.5 kg and a standard deviation of 18.7 kg (76.7+/− 11.6 kg, for axitinib), truncated between 36 and 185 kg. Baseline tumor size followed a lognormal distribution, with a mean of 182.7 mm and a standard deviation of 134.2 mm, truncated between 29 and 822 mm.

### Baseline Simulations

Following current guidelines, initial simulations of sunitinib and axitinib were performed using a fixed dose at 37.5 mg daily or 5 mg twice per day (b.i.d.), respectively [Pfizer, Inc. SUTENT^®^ (Sunitinib malate), 2006; Pfizer, Inc. INLYTA^®^ (Axitinib), 2012]. No dose escalations were allowed in the base simulations. Dose reductions were allowed in the event of unacceptable toxicity (≥Grade 3, or ≥Grade 2 for fatigue and HFS), following National Cancer Institute Common Toxicity Criteria (CTCAE) v3.0. To replicate a clinical scenario, monitoring of ANC, dBP, and platelet count occurred solely on days 15, 29, 43, 57, 85, 113, and once every 12 weeks thereafter. Fatigue and HFS were assumed to be spontaneously reported side effects that were monitored on a daily basis.

Dose adjustments were simulated with a discrete number of possible sunitinib and axitinib doses. Available sunitinib doses ranged from 0 to 75 mg of sunitinib once daily, in 12.5 mg increments. For axitinib the available doses were 0, 2, 5, 7, or 10 mg b.i.d. In the event of primary Grade 3 (or Grade 2 for HFS and fatigue) toxicity, the drugs were withheld until ≤ Grade 1 toxicity. Hereafter, the drugs were resumed at the initial dose. In case of repeated, i.e. > 1 occurrence of Grade 3 (or Grade 2 for HFS and fatigue), or severe Grade 4 (or Grade 3 for HFS and fatigue) toxicity, the drugs were withheld until ≤ Grade 1 toxicity, and thereafter resumed at one dose level reduction.

### Simulation of High-Dose Treatment

The influence of intermittent high-dose sunitinib or axitinib was evaluated by comparing once weekly (QW), and once every 2 weeks (Q2W) administration, to standard continuous dosing. For these simulations, the available pool of sunitinib doses was extended with doses of 100–700 mg in 100 mg increments. The initial sunitinib dose was fixed to 300 mg for QW and 700 mg for Q2W, with possible dose reduction in the event of AEs (Rovithi et al., 2016). The pool of available axitinib doses was extended with doses of 10–80 mg in 10 mg increments, and 80–140 mg in 20 mg increments, for QW and Q2W dosing regimens, respectively. Axitinib starting doses were at 70 mg for QW, or 140 mg for Q2W, with possible dose reductions in case of AEs.

### Identification of Biomarker Cut-Off Points

Different threshold values for each biomarker were evaluated in order to divide biomarker findings into “adjustment” and “no adjustment” dosing decisions. The influence of each biomarker cut-off point was simulated (n = 1,000) to predict population survival and the development of AEs. Individual predicted values of the biomarker (“IPRED”) were used to evaluate the direct effect of dose interventions, i.e. the residual error resulting in a difference between the clinically measured value (“Y”) from the “true” value IPRED, was omitted. Threshold values with similar OS outcomes across TDM, BAD, and TAD were selected in order to ensure that the biomarker-based dose-adjustment approaches could be compared with respect to the development of AEs and cost-effectiveness. If differing OS values would be utilized, the difference between biomarkers could otherwise be caused by discrepancies in biomarker cut-off values. The predicted OS when using the sunitinib TDM threshold was used as reference to ensure equal threshold selections also in the BAD and TAD simulation frameworks.

Cut-off values between 0 ng/ml and 80 ng/ml (sunitinib plus SU12662, or axitinib) were evaluated for TDM of C_trough_. Since most axitinib exposure-response analyses have focused on the relationship between AUC and response (Verheijen et al., 2017), the correlation between axitinib AUC and C_trough_ (IPRED) was firstly evaluated using Pearson's correlation coefficient to warrant the adaptation of C_trough_ in TDM scenarios.

For TAD based on neutrophils, values between 1 × 10^9^ cells/L and 5 × 10^9^ cells/L were evaluated as cut-offs. Similarly, for BAD based on sVEGFR-3 a range between 5 and 80%, in addition to 0%, decrease from individual sVEGFR-3 baseline was assessed. For TAD based on dBP, cut-off values in the range 2.5 to 40%, in addition to 0%, increase from individual baseline values were evaluated. Based on the biomarker threshold-survival plots, cut-off values were selected for each biomarker that constrained the total number of developed AEs and resulted in equal survival for TDM, TAD, and BAD.

### Model-Based Forecasts

The accuracy of clinical sample measurements (“Y”) was evaluated by taking the ratio between the observed change measured in clinical samples at week 8 (BIOMARKER_Y_) and the true biomarker change (e.g. Eqs. 1–3) at week 8 (BIOMARKER_IPREDtrue_) (Eq. 4). The accuracy of model-based estimations of biomarker changes (BIOMARKER_IPREDest._) was evaluated under different monitoring durations and sampling frequencies. The simulation-estimation procedure was performed using the proseval command (prospective evaluation; Pearl-speaks-NONMEM [PsN])^1^ in NONMEM (version 7.3.0) (Beal et al., 1989; Keizer et al., 2013). The function enables automated performance evaluation of maximum *a posteriori* prediction (MAP) Bayesian estimations based on varying amount of information, i.e. using an increasing number of data points per individual (Abrantes et al., 2019). Based on the dataset of n observations per individual, the command initiates estimations based on the first observation in each individual and runs successive estimations adding one additional observation in each round (in the order of sampling time), until the final number of n observations is reached. As such, the proseval command facilitates the process of determining the influence of observation numbers on individual biomarker value estimation.

In order to determine the influence of monitoring durations and frequencies on the model-based estimations, daily BIOMARKER_Y_ values were simulated for ANC, dBP, sVEGFR-3, and drug plasma concentration (n = 1,000) using the “true” individual values IPRED_true_ with an added random residual error. Datasets containing different numbers of biomarker measurements were generated through changes in (1) monitoring duration (week 0 to week 8) and (2) sampling frequencies (daily, weekly, and biweekly). Based on these outputs, individual parameters were obtained (IPRED_est_) within the corresponding population pharmacokinetic and pharmacodynamic models, using maximum-a-posteriori estimation. The final BIOMARKER_IPREDtrue_ were simulated using the IPRED_true_ values and compared to the individual BIOMARKER_IPREDest_ values simulated with IPRED_est_. Accuracy of model estimations were computed by taking the ratio of the forecasted estimated biomarker changes (BIOMARKER_IPREDest._) and true biomarker changes (BIOMARKER_IPREDest_) from baseline at week 8 (Eq. 5).

(4) $$Accuracy_{clinical_{sample}}=BIOMARKER_{Y}÷BIOMARKER_{IPREDtrue}$$

(5) $$Accuracy_{IPREDest}=BIOMARKER_{IPREDest}÷BIOMARKER_{IPREDtrue}$$

The capacity of each method to give rise to correct dose recommendations was further summarized by means of its sensitivity and specificity to inform correct dosing decisions, using the determined biomarker cut-off values. Here sensitivity represents the percentage of times dose increments were recommended at t = 1,344 h (e.g. day 57/week 8) according to the estimated (BIOMARKER_IPREDest_) or measured (BIOMARKER_Y_) changes in biomarker value, compared to the number of times that the “true” biomarker change (BIOMARKER_IPREDtrue_) would require dose increase. Specificity represents the percentage of times dose increments were not recommended at week 8 according to the estimated (BIOMARKER_IPREDest_) or measured (BIOMARKER_Y_) changes in biomarker value, compared to the number of times that the “true” biomarker change (BIOMARKER_IPREDtrue_) obliges no dose alternations.

### Simulating Cost-Effectiveness

Cost-effectiveness of biomarker-based dose individualization was determined *via* differences in absolute costs and quality adjusted life years (QALYs) between standard and individualized treatments. Information regarding treatment costs and hospital expenses of sunitinib therapy were added to the simulation frameworks to analyze the expenditures, as seen in previous work (van Hasselt et al., 2015; Zuidema et al., 2019). Costs of regular follow-up procedures, including medical visits, laboratory investigation, and radiology tests (following clinical guidelines for treatment of metastatic and unresectable GIST), were gathered from the Dutch Healthcare Authority (NZa^2^, accessed 12 October 2019). Expenditures related to the development of AEs were collected from (Mickisch et al., 2009), summarizing the costs of each AE in different countries. Because of the absence of Dutch data, the German costs were employed for calculating expenses related to AEs, as the German healthcare system is most compatible to the Dutch one (Zuidema et al., 2019). Drug-related costs were computed by multiplying the price of each sunitinib dose category by the cumulative number of capsules. Prices of each capsule were collected from the Dutch National Health Care Institute (Zorginstituut Nederland^3^, accessed 12 October 2019).

To account for potential differences in health status between the biomarker schedules, cost-effectiveness was calculated using both gained life-years and QALYs. In contrast to gained life-years, QALYs combine information regarding both increase in survival time as well as the quality of health during these years, which may be affected by the presence of AEs or changes in disease status. The number of QALYs for a given individual is commonly calculated by multiplying the total number of life-years-gained by the quality of life in these years, generally scored between 0 (equivalent to death) and 1 (equivalent to perfect health) according to the EuroQol- 5 Dimension (EQ-5D) questionnaires (Whitehead and Ali, 2010). In cost-effectiveness analysis, such scores are known as “utility values.” To quantify the overall health quality score at a given moment, utility values of relevant factors should be taken into consideration. For sunitinib, utility values related to baseline status, disease progression, and the development of AEs were here gathered from literature (Paz-Ares et al., 2008; Beauchemin et al., 2016; Liviu Preda and Galieta Mincă, 2018). Individual utility values (UV_i_) at each time point (UV_ij_) were computed by multiplying relevant UV_i_'s under disease and the predicted Grades of AE (0–4) (Eq. 6). Individual QALYs (QALY_i_) were then calculated by dividing the sum of daily UV_ij_ values by days between the start and end of treatment (t_treatment_), and multiplying by the amount of life-years-gained per individual (LYG_i_) (Eq. 7).

(6) $$UV_{ij}=UV_{neutropenia,ij}\times UV_{trombocytpenia,ij}\times UV_{hypertension,ij}\times UV_{fatigue,ij}\times UV_{HFS,ij}\times UV_{diseasestatus,ij}$$

(7) $$QALY_{i}=LYG_{i}\;x\;sum(UV_{ij})/t_{treatment}$$

Using the established biomarker threshold values, the influence of each sunitinib biomarker was simulated for a virtual patient population (n = 1,000) over a 5-years time horizon, as recommended for cost-effectiveness analysis (Kim et al., 2017) on gained life-years, QALYs, development of AEs, and treatment costs. Due to that AE models were not available for all common AEs during axitinib treatment, this analysis was not possible for the axitinib framework.

## Results

### High-Dose Intermittent Administration

Based on the simulation output, OS of the GIST population at week 102 was predicted to be higher under sunitinib standard CD dosing (37.5 mg), compared to QW (300 mg) and Q2W (700 mg) dosing (**Table 1**). The frequency of high-grade AEs was predicted to be pronouncedly lower for the high-dose schedules, with a relatively low frequency of neutropenia, HFS, and thrombocytopenia for QW, Q2W dosing, compared to CD dosing. The incidence of fatigue was comparable between the QW, Q2W, and CD dosing schedules. Frequency of diastolic hypertension was substantially higher under QW and QW dosing, compared to CD dosing. For axitinib, OS of the mRCC population at week 102 was predicted to be higher under standard (5 mg b.i.d.) dosing, compared to QW (70 mg) and Q2W (140 mg) dosing.

**Table 1:** Efficacy and safety under sunitinib CD (37.5mg), QW (300 mg), and Q2W (700 mg) dosing for GIST; Efficacy under axitinib b.i.d. (5 mg), QW (70 mg), and Q2W (140 mg) dosing for mRCC.

	Survival GIST, at 102 weeks (%)	Neutropenia (max %)	Thrombocytopenia (max %)	HFS (max %)	Fatigue (max %)	Diastolic hypertension (max %)	Survival mRCC, at 102 weeks (%)
b.i.d./CD	48.2%	3.7%	8.4%	10%	1.9%	1%	65.2%
QW	42.6%	0%	0%	1%	1.9%	4.8%	50.7%
Q2W	39.6%	0%	0.1%	1.1%	2.3%	17.1%	48.5%

CD, continuous daily; QW, once weekly; Q2W, once every two weeks.

### Biomarker Threshold Values

**Figure 2:**
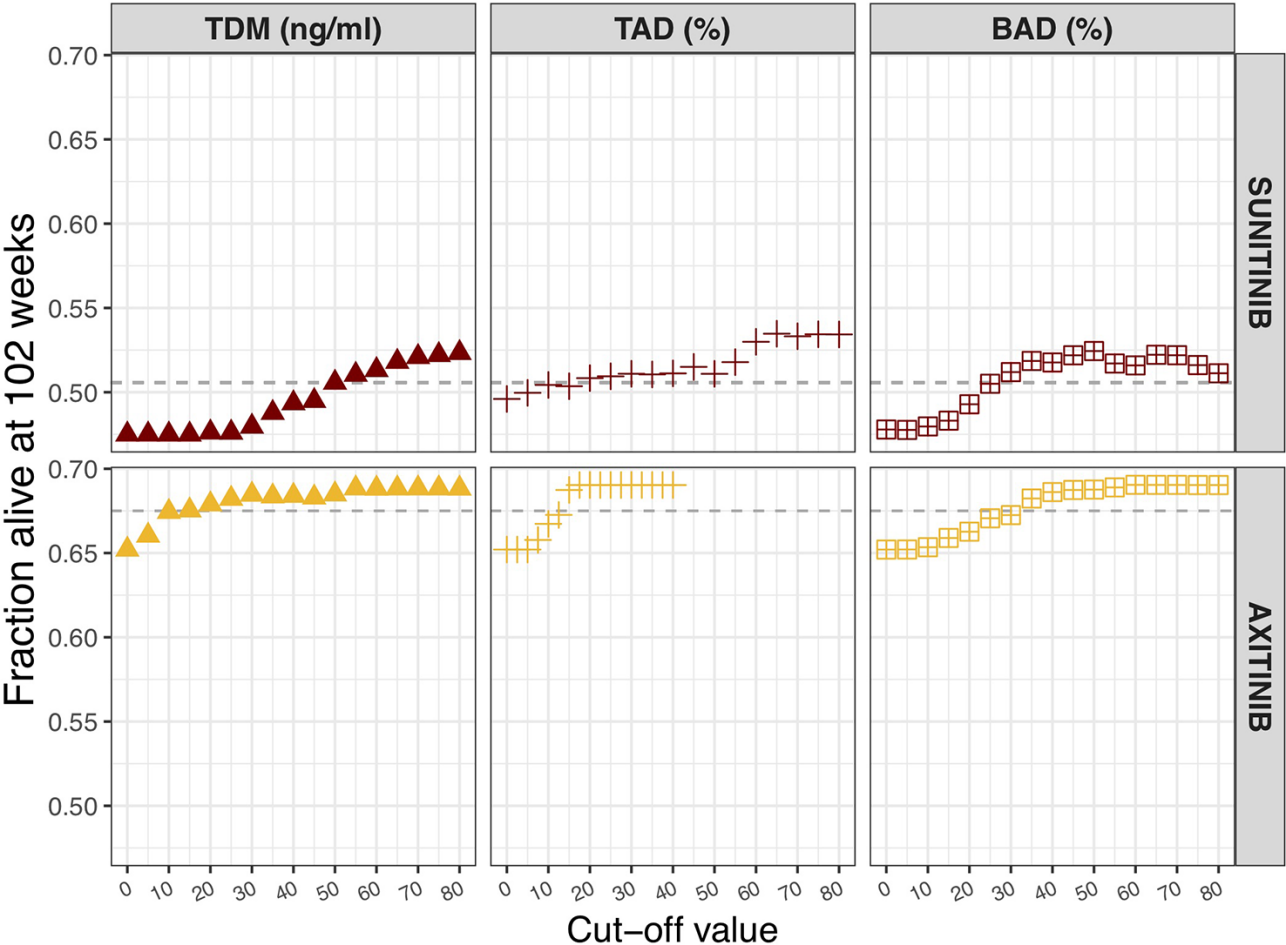
Influence of biomarker threshold values on population overall survival at 102 weeks. For TDM of sunitinib (sunitinib and SU1266) and axitinib C_trough,ss_ values of 0 to 80 ng/ml were assessed. For TAD based on neutrophils, a relative decrease from 0 to 80% from the population baseline value (5 × 10^9^ cells/L) was evaluated. In case of TAD based on dBP, relative increase of 0 to 40% from individual baseline value was chosen. For BAD of sVEGFR-3, relative decrease from 0 to 80% of individual baseline value was assessed. *BAD, biomarker-adjusted dosing; TAD, toxicity-adjusted dosing (ANC for sunitinib; dBP for axitinib); TDM, therapeutic drug monitoring*.

The influence of different biomarker threshold values is visualized against changes in OS (**Figure 2**). The selected sunitinib TDM cut-off value of 50 ng/ml, resulted in 50.5% OS at 102 weeks. For ANC, a 20% decrease from population baseline (e.g. from 5 to 4 × 10^9^ cells/L) resulted in the same OS rate. For BAD, a cut-off value of 25% decrease in sVEGFR-3 from individual baseline was predicted to equal the threshold of 50 ng/ml sunitinib threshold in terms of OS.

Pearson's correlation coefficient between axitinib AUC and C_trough_ (IPRED) was found to be 0.679 (p < 0.001, n = 1,000). An axitinib TDM cut-off value of 10 ng/ml was selected, resulting in 67.5% OS at 102 weeks. The same OS rate was predicted for an on average 12.5% increase in dBP and a 30% decrease in sVEGFR-3, from individual baselines.

### Biomarker Forecasts

**Figure 3:**
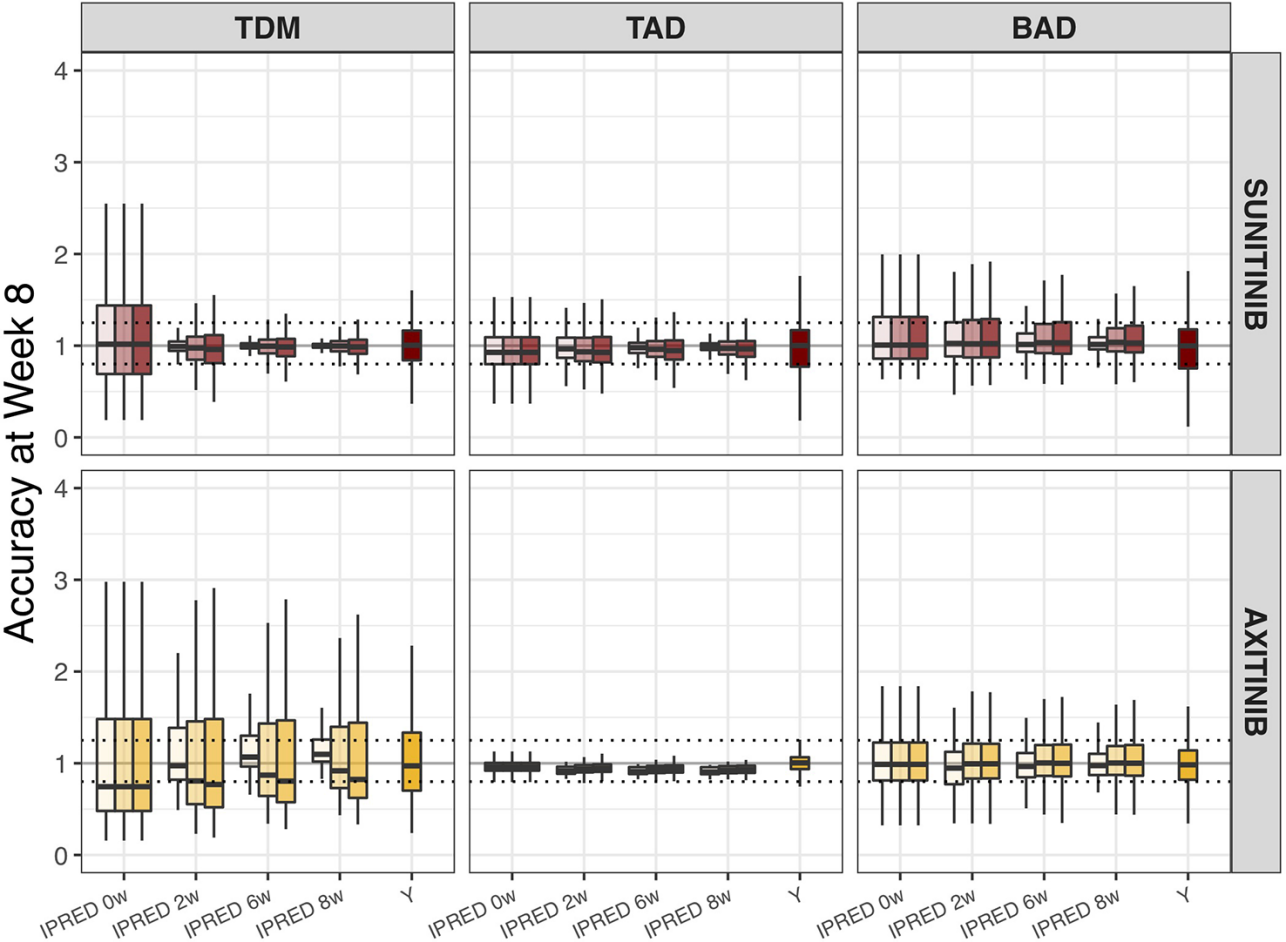
Accuracy of biomarker forecasts (IPRED) versus clinical sample-based methods (Y). Accuracy in the simulated population (n = 1,000) as illustrated by the distribution of model-based biomarker forecasts (BIOMARKER_IPREDest_) or directly measured sample values (Y) under continuous bidaily dosing of axitinib at 5 mg or daily dosing of sunitinib at 37.5 mg. Accuracy = [Predicted (BIOMARKER_IPREDest_) or Measured (Y) biomarker change/Actual biomarker change (BIOMARKER_IPREDtrue_)]. Each plot represents forecasts accuracies at week 8 under different monitoring durations (0, 2, 4, 6, or 8 weeks; IPRED on x-axis) and sampling frequencies (daily [light], weekly [medium], or biweekly [dark]; color shades). For directly measured clinical samples (Y on x-axis) each plot represents the accuracy to capture the true biomarker change at week 8, using one measurement at baseline and one at week 8. Solid black line means 100% accuracy and represents the state at which predicted biomarker change = actual biomarker change. Dashed horizontal lines represent the range wherein the predicted biomarker change falls within the 80–125% range of the true biomarker change. The ends of each box represent the population 25th and 75th percentiles and whiskers the 2.5th and 97.5th percentiles, respectively. Median is represented by the line that cuts through each box in the plot. *BAD, biomarker-based dosing; TAD, toxicity-adjusted dosing (ANC for sunitinib; dBP for axitinib); TDM, therapeutic drug monitoring*.

The accuracy of model-based biomarker forecasts compared to using observed clinical biomarker measurements is depicted in **Figure 3**. The accuracy plots represent the capacity of each method to predict biomarker value at t = 1,344 h (e.g. day 57/week 8). For most biomarkers, model-based predictions demonstrated higher accuracy than direct clinical measurements, particularly when > 1 measurement informed the Bayesian feedback estimation. It is important to notice that IPRED = 0 was estimated solely on the measurement made at t = 0, which adds to the baseline value of the ANC, dBP, and sVEGFR-3 biomarkers. For the TDM biomarker, however, this represents a sampling moment antecedent to start of drug administration (e.g. a concentration of 0) and IPRED = 0 is therefore representing the population distribution prior to individual knowledge.

**Figure 4:**
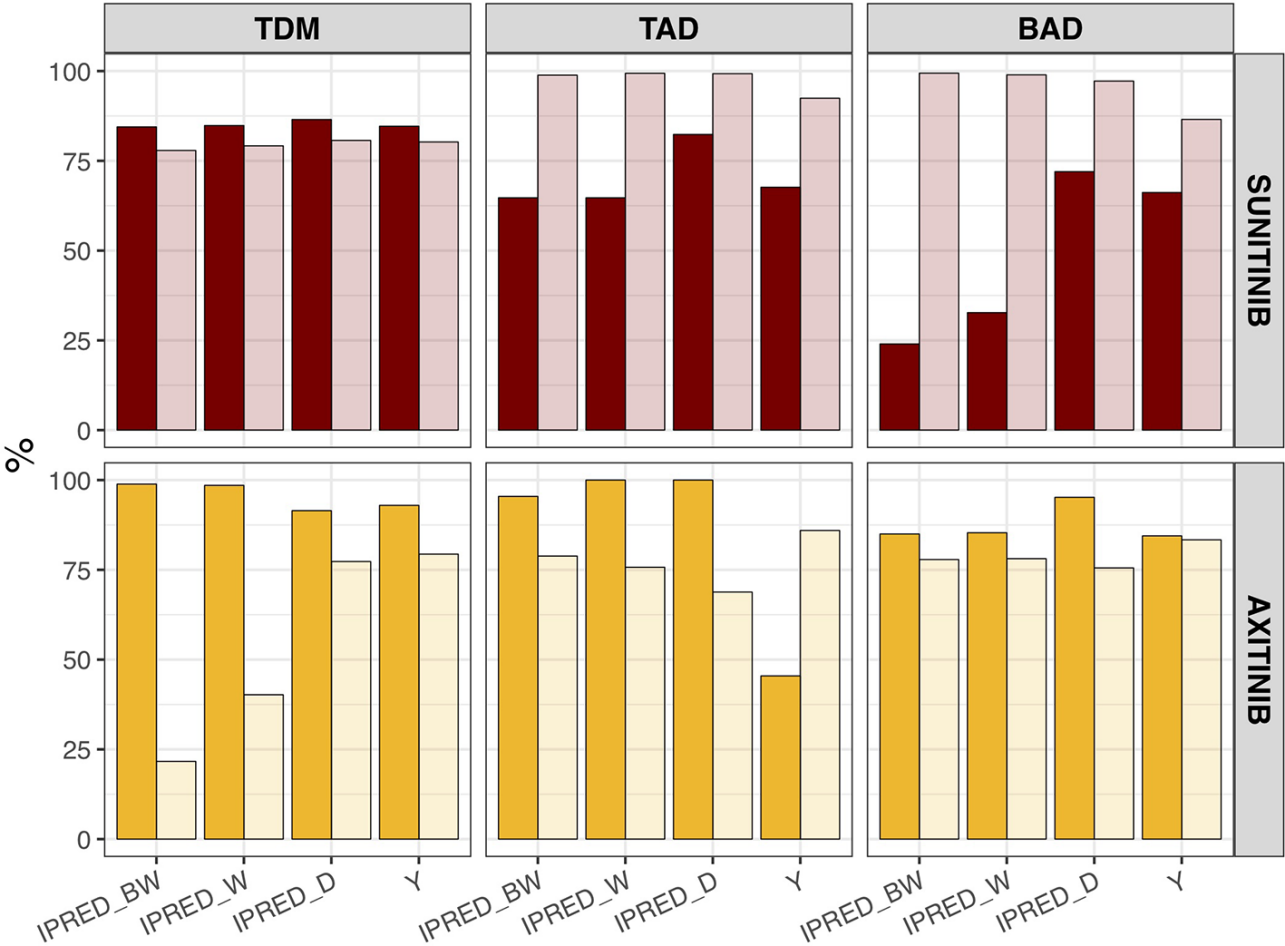
Sensitivity and specificity of dose decisions based on biomarker forecasts (IPRED) *versus* clinical sample-based methods (Y). Percentage of correct dose recommendations in the simulated population (n = 1,000) in model-based biomarker forecasts (IPRED) or directly measured sample values (Y) under continuous bidaily dosing of axitinib at 5 mg or daily dosing of sunitinib at 37.5 mg. Each plot represents the dose recommendation at week 8 under different sampling frequencies (biweekly [IPRED_BW], weekly [IPRED_W] or daily [IPRED_D]; on x-axis). For directly measured clinical samples (Y on x-axis) each plot represents the dose recommendation at week 8, following one measurement at baseline and one at week 8. Sensitivity = [amount of times dose increases/amount of times dose should have been increased according to biomarker value] × 100%. Sensitivity is represented by the dark shaded bars. Specificity = [amount of times dose did not increase/amount of times dose should not have been increased according to biomarker value] × 100%. Specificity is represented by the light shaded bars. *BAD, biomarker-based dosing; TAD, toxicity-adjusted dosing (ANC for sunitinib; dBP for axitinib); TDM, therapeutic drug monitoring*.

The percentage of correct dose decisions that was made at t = 1,344 (e.g. day 57/week 8) following model-based biomarker forecasts (IPRED) or clinical biomarker measurements (Y) is summarized in **Figure 4**. Here the sensitivity (e.g. percentage of times the method correctly states dose increase should occur) is demonstrated for forecasts based on biweekly, weekly, and daily measurements (following 8 weeks of measurements), as well as for clinical biomarker samples (following one sample at t = 0 and one sample at t = 1,344 h). Specificity (e.g. percentage of times the method correctly states dose increase should not occur) is depicted in a similar manner. Both sensitivity and specificity vary between different biomarkers and prediction method, e.g. model-based prediction *versus* clinical biomarker measurements, however, model-based estimations generally improve with an increasing measurement frequency.

### Cost-Effectiveness Analysis

Based on our sunitinib model we estimated median OS at 1.71, 1.80, 1.90, and 2.16 years for the fixed, TDM-based, ANC-based, and sVEGFR-3-based dosing, respectively (**Table 2**). The cost per additional life-year was highest for TDM-based (€115 433), when compared to ANC-based (€71 458) and sVEGFR-3-based (€25 340) dosing. Cost per additional QALY was found to be substantially lower for s-VEGFR-3 (€36 784), compared to TDM-based (€173 150) and ANC-based (€104 438) dosing.

**Table 2:** Sunitinib biomarker cost-effectiveness analysis, median values.

	Life-years (95% CI)	QALYs (95% CI)	Cumulative AEs	Expenditures, euro (95% CI)	Expenditures per life-year, euro (95% CI)	Expenditures per QALY, euro (95% CI)
**Fixed dosing**	1.71 (1.60–1.86)	1.16 (1.08–1.27)	1373	49360 (47651–51069)		
**TDM-based dosing**	1.80 (1.65–1.96)	1.22 (1.12–1.33)	1564	59749 (57326–62172)	115433 (88511–142356)	173150 (132767–213533)
**ANC-based dosing**	1.90 (1.72–1.98)	1.29 (1.17–1.34)	1723	62937 (60538–65337)	71458 (58832–84089)	104438 (85985–122900)
**sVEGFR-3-based dosing**	2.16 (2.02–2.32)	1.47 (1.37–1.58)	1680	60763 (58335–63190)	25340 (19944–30733)	36784 (28952–44613)

CI, confidence interval; QALY, quality-adjusted life-years.

## Discussion

Two simulation frameworks were created that allowed for survival and safety analysis of different (I) treatment schedules and (II) biomarker-guided dose individualization options for axitinib in mRCC and sunitinib in GIST. Moreover, (III) model-based dose individualizations were weighted against current sample-based approaches. For sunitinib, where relevant AE models were available, the framework was additionally employed to (IV) assess the cost-effectiveness of sunitinib therapy under each biomarker option. The abovementioned approaches serve as a methodological example of how model-based frameworks can be applied to assess schedule adjustments, select biomarkers for dose adaptations, and perform pharmacoeconomical analyses.

Simulations from the axitinib and sunitinib frameworks suggest that QW or Q2W high-dosing at the cumulative weekly standard CD dose might result in lower OS in patients with mRCC and GIST, compared to CD dosing. With the exception of diastolic hypertension under sunitinib, the development of AEs was however lower or similar under QW or Q2W high-dose compared to CD dosing, which complies with clinical findings (Rovithi et al., 2018). A possible explanation for this finding resides in the relationship between the axitinib and sunitinib exposure and the development of AEs, which is directly or indirectly defined by Emax equations, with exception of the sunitinib AUC-dBP relationship that is defined by a linear relationship. The adverse effect caused by drug exposure is consequently expected to asymptote to a maximum effect at higher values, thus the high dose regimens is overall resulting in less toxicity over time given that the longer dosing interval allows for washout. The distinction between the development of AEs will be determined by the longevity of drug effect; e.g. CD will cause more adverse effects compared to QW or Q2W schedules.

It is important to note that extrapolation outside the original dosing schedule, that the models were built on, result in more uncertain predictions and clinical study data would need to confirm the findings and/or serve the basis for refining the models. Since current models were based on frequent, low-dose schedules of axitinib and sunitinib, they may not capture pleiotropic drug effects that can arise at intermittent, high doses. Although the models appear to capture the development of AEs in comparison to clinical data, it is possible that the linear and E_max_ equations that are defined using lower dosages may not accurately represent the relationships between drug exposure and effect that occur at higher dosages. As such, schedule adjustments will often necessitate an iterative endeavor of prediction, data generation, and confirmation (EMA extrapolation framework ^4^, accessed 15 November 2019).

Model-based forecast values of biomarkers on >1 clinical measurement provided higher accuracy than most single direct clinical sample measurement values, particularly for dBP under axitinib (**Figure 3**). Interestingly, previous studies found model-based estimations to have low predictive accuracy for changes in dBP under sunitinib (Centanni et al., 2018). This inconsistency may be due to the presence of inter-individual variability on two additional parameters (i.e. drug effect and dBP turnover rate) and the existence of a combined additive and proportional error instead of just a proportional error. Extrapolation of biomarkers between treatment indications or members of a drug class should therefore be made with caution. In a similar manner, the influence of model-based estimations and clinical sample measurements on the percentage of correct dosing decisions differs between the different biomarkers and drugs (**Figure 4**). For axitinib, TDM-guided dosing using a model-based method appears to overestimate the number of dose increases that should occur (i.e. low specificity) under the more sparsely sampled (e.g. biweekly) data, compared to just evaluating the clinical measurement at week 8. This is likely due to the initial relative underestimation in drug concentration that is seen in **Figure 3**. A similar pattern is observed for BAD of sunitinib, where the sensitivity of the model-based estimations is lower than the clinical biomarker measurement when using sparse sampling (**Figure 4**), due to initial overestimations of biomarkers changes (**Figure 3**). For the remaining biomarkers, model-based estimations with sparse data provided either an equal or improved percentage of correct dosing decisions, as compared to clinical sample measurements. Importantly, it is expected that model-based estimations are capable of providing predictions close after treatment initiation, whereas clinical samples can only inform dosing decisions once steady-state has been reached (Svensson et al., 2019).

Limitations related to the use of model-based dose individualization for TKIs mainly involve the complexity and computational power related to the model building process, and consequently the availability of such models and software to have a direct implementation at the point of care (van Beek et al., 2019). In addition, since the current models are based upon rich, but often more selected, datasets derived from clinical trials they might require further adjustments to represent the parameter distributions of the entire patient population (Keizer et al., 2018). Advantages of model-based dose individualization of TKIs reside in the (i) capacity to take previous sample observations and patient-specific characteristics into account, (ii) ability to consider a modified dosing history, (iii) flexibility in when to take measurements and finally (iv) capability to predict future effects following dose adjustments.

Cost-effectiveness analysis of biomarkers suggest that sunitinib dose individualization based on sVEGFR-3 results in highest increase in median OS and lowest costs per QALY. Following current clinical expenses, a median increase of €36 784 per QALY was determined. Using the threshold of €80 000 and 500,000 SEK (~€57,000) per gained QALY, this would entail that sVEGFR-3 based dosing can be regarded as a cost‐effective intervention in the Netherlands and Sweden, respectively (Vallejo-Torres et al., 2016). The cost-effectiveness of ANC-based and TDM-based dosing appears low, at €104 438 and €173 150 per gained QALY, respectively. Because biomarker threshold values were not set with the primary purpose of increasing OS, alternative biomarker threshold values may optimize cost-effectiveness of ANC-based and TDM-based dosing. Since the correlation between axitinib AUC and C_trough_ is not perfect (r = 0.679), it is recommended to perform an exposure-response analysis to further evaluate the correlation between C_trough_ and treatment outcome and determine the relevance of C_trough_ to guide axitinib dose individualization.

The main disadvantage of cost-effectiveness analysis using a model framework resides in the initial creation of mechanistic models, which demands a significant investment of time and effort. As such, it might primarily appear less desirable. With increasing necessity for model-based analysis in regulatory drug approval it is, however, expected that the required models will become readily available for future developed compounds (Lee et al., 2011). Due to its mechanistic nature it is additionally expected that the predictions made by a model framework are more biologically plausible and therefore better resemble reality (van Hasselt et al., 2015). Moreover, the flexibility of the current model framework the exploration of alternative dosages requires limited additional effort.

To conclude, a model-based framework that includes PK, AEs, circulating biomarkers, tumor growth dynamics, and their relation to OS can be employed to assess alternative treatment schedules and dose-individualization approaches. As more clinical data is collected, each framework can become further extended to reflect the “real-world” population, and to meet novel clinical needs (Keizer et al., 2018). For models in which patient-specific factors (i.e. “covariates”) determine treatment effect, the impact of subpopulations can be simulated and compared to the overall population. As such, the framework provides a flexible approach to evaluate the safety, efficacy, and cost-effectiveness of treatments. In addition to simulations, future efforts should focus on verifying the added value of model-based dose adjustments.

## Data Availability Statement

The datasets generated for this study are available on request to the corresponding author.

## Author Contributions

MC performed the analysis and simulations. Both LF and MC contributed to the final version of the manuscript. LF supervised the project.

## Funding

The study was supported by the Swedish Cancer Society. CAN 2017/626.

## Conflict of Interest

The authors declare that the research was conducted in the absence of any commercial or financial relationships that could be construed as a potential conflict of interest.

## Footnotes

1 https://github.com/UUPharmacometrics/PsN/releases; proseval_userguide.pdf

2 https://www.nza.nl/english

3 www.farmacotherapeutischkompas.nl

4 https://www.ema.europa.eu/en/documents/presentation/presentation-ema-extrapolation-framework-christoph-male_en.pdf
