# Supplementary material for: Model-Based Biomarker Selection for Dose Individualization of Tyrosine-Kinase Inhibitors
Source: Front Pharmacol. 2020 Mar 12;11:316. doi: 10.3389/fphar.2020.00316 (PMC7080977; doi:10.3389/fphar.2020.00316)
Supplement: Supplementary file 1 [file Table_1.docx]

**Table S1.1** Parameter estimates for pharmacokinetic models sunitinib and active metabolite SU12662 (Yu et al)

| Parameter | Estimate (RSE,%) | IIV, %CV (RSE,%) |
| --- | --- | --- |
| Sunitinib |  |  |
| Ka (h-1) | 0.34 (10.8) | - |
| CL (l·h-1) | 35.7 (5.7) | 33.9 (12.0) |
| Vc (l) | 1360 (6.0) | 32.4 (10.6) |
| Qh (l·h-1) | 80 FIX | - |
| Vp (l) | - | - |
| σprop | 0.06 (13.5) | - |
| SU12662 |  |  |
| CL (l·h-1) | 17.1 (7.4) |  |
| Vc (l) | 635 (13.1) |  |
| Qi (l·h-1) | 20.1 (32.6) | - |
| Vp (l) | 388 (14.9) | - |
| σprop | 0.03 (14.1) | - |
| Correlations |  |  |
| ρ(CLsunitinib, CLSU12662) | 0.53 | - |
| ρ(Vc,sunitinib, Vc,SU12662) | 0.48 | - |
| ρ(CLSU12662, Vc,SU12662) | 0.45 | - |

CL = clearance; CV = coefficient of variation; IIV = inter-individual variability; *K*a = absorption rate constant; Qh = hepatic blood flow; Qi = intercompartment clearance; RSE = relative standard error; *V*c = volume of distribution of central compartment; *V*p = volume of distribution of peripheral compartment; ρ = correlation coefficient; σprop = proportional residual error.

**Table S2.1** Parameter estimates for biomarker models sVEGFR-3 and sKIT (relative SEs, %) (Hansson et al)

|  | **sVEGFR-3** | | **sKIT** | |
| --- | --- | --- | --- | --- |
| Parameter (unit) | Estimate | IIV CV, % | Estimate | IIV CV, % |
| BM0 (pg/ml) | 63,900 (3.7) | 43 (12) | 39,200 (3.0) | 50 (8.1) |
| MRT (days) | 16.7 (7.2) | 24 (14)^a^ | 101 (8.8) | 27 (25) |
| Hill parameter | - | - | — | - |
| IC50 (mg·h/l) | 1.00 (5.3)^b^ | 63 (16) | 1.00 (5.3)^b^ | 240 (14) |
| DPslope (/month) | - | - | 0.0261 (29)^c^ | 172 (13) |
| Residual error (%) | 21.9 (10) | - | 22.6 (8.4) | - |
| Residual error (pg/ml) | - | - | - | - |

BM0 = biomarker level at baseline; CV = coefficient of variation; DPslope = disease progression slope; IC50 = daily sunitinib area under the curve resulting in half of the maximum drug effect; IIV = interindividual variability; MRT = mean residence time; sKIT = soluble stem cell factor receptor; sVEGFR = soluble VEGF receptor; VEGF = vascular endothelial growth factor.
^a^Common IIV parameter. ^b^Common IC50 parameter. ^c^Common DP_slope_ parameter.

**Table S3.1** Parameter estimates for adverse event models neutropenia, diastolic hypertension and thrombocytopenia (relative SEs, %) (Hansson et al)

| Parameter | Estimate | RSE, % | IIV CV, % | RSE, % |
| --- | --- | --- | --- | --- |
| Neutropenia model | |  |  |  |
| ANC_0_ (⋅109/l) | 3.69 | 6.9 | 42 | 5.6 |
| MTT (hours) | 248 | 36 | 17 | 19 |
| ANC Emax | 0.520 | 9.1 | 13 | 36 |
| ANC EC50 (pg·hour/l) | 0.552 | 17 | 46 | 16 |
| γ | 0.362 | 7.4 | - | - |
| Residual error^a^ | 0.406 | 4.3 | - | - |
| Blood pressure model | |  |  |  |
| dBP_0_ (mmHg) | 71.8 | 1.0 | 12 | 6.7 |
| MRT (= 1/kout) (hours) | 361 | 17 | 83 | 12 |
| dBPslope (l/mg·hour) | 0.119 | 9.4 | 65 | 11 |
| Residual error (mmHg) | 6.24 | 16 | - | - |
| Residual error (%) | 6.97 | 24 | - | - |
| Thrombocytopenia model |  |  |  |  |
| ATC_0_ (⋅109/l) | 323 | 2 | 32.4 | 10 |
| MTT_ATC_ (hours) | 172 | 4 | 17.5 | 19 |
| ATC Emax | 0.497 | 33 | - | - |
| ATC EC50 (pg·hour/l) | 10.3 | 35 | 60.8 | 23 |
| γ_ATC_ | 0.0923 | 9 | - | - |
| Residual error ^b^ | 0.268 | 7 | - | - |

ANC_0_ = baseline absolute neutrophil count; ATC_0_ = baseline absolute trombocyte count; CV = coefficient of variation; dBP0 = baseline diastolic blood pressure (dBP); dBPslope = parameter relating drug exposure to the change in dBP; IIV = interindividual variability; MRT = mean residence time; MTT = mean transit time; MTT_ATC_ = mean transit time thrombocyte model; RSE = relative standard error; γ = feedback factor; γ_ATC_ = feedback factor in thrombocyte model.

^a^ Residual error (additive) on Box–Cox-transformed scale. ^b^Residual error (additive) on log-transformed scale.

**Table S4.1** Parameter estimates for hand-foot syndrome and fatigue models (relative SEs, %) (Hansson et al)

|  | HFS model | | Fatigue model | |
| --- | --- | --- | --- | --- |
| Parameter | Estimate | RSE, % | Estimate | RSE, % |
| B1\|0 | −10.4 | 11 | −5.85 | 3.0 |
| B2\|0 | −0.974 | 13 | −1.14 | 7.8 |
| B≥3\|0 | −1.59 | 19 | −1.60 | 14 |
| Slopex\|0 | −8.00 | 14 | −1.93 | 22 |
| ωx\|0 | 3.07 | 67 | 1.06 | 20 |
| B1\|1 | 2.29 | 14 | 2.63 | 10 |
| B2\|1 | −9.53 | 5.0 | −10.7 | 3.1 |
| B≥3\|1 | −1.33 | 24 | −1.77 | 22 |
| Slopex\|1 | −6.00 | 18 | −4.62 | 17 |
| ωx\|1 | 0.902 | 54 | 1.25 | 30 |
| B1\|2 | 3.04 | 15 | 2.86 | 12 |
| B2\|2 | −0.747 | 14 | −0.427 | 20 |
| B≥3\|2 | −9.09 | 5.1 | −11.6 | 5.0 |
| Slopex\|2 | −3.23 | 43 | −4.64 | 22 |
| ωx\|2 | 0.270 | 118 | 1.30 | 24 |
| B1\|>3 | 3.4 | 21 | 3.06 | 23 |
| B2\|>3 | −1.65 | 23 | −0.090 | 115 |
| B≥3\|≥3 | −0.453 | 37 | −0.636 | 33 |
| Slopex\|≥3 | −4.75 | 32 | −3.32 | 51 |
| ωx\|≥3 | NE | NE | 0.841 | 71 |

Bb|a, intercept for the probability of transition from grade a to grade b; HFS, hand–foot syndrome; NE, not estimated; RSE, relative standard error; Slopex|b, parameter relating soluble vascular endothelial growth factor receptor (sVEGFR)-3 to the probability of the severity score x given the previous score b; ωx|0, interindividual random variability.

**Table S5.1** Parameter estimates for tumor growth inhibition, dropout, and survival models (relative SEs, %) (Hansson et al)

| **Tumor inhibition model** | | | **Dropout model** | | **Survival model (sVEGFR-3)** | | **Survival model (AE)** | |
| --- | --- | --- | --- | --- | --- | --- | --- | --- |
| Parameter (unit) | Estimate | IIV CV (%) | Parameter (unit) | Estimate | Parameter (unit) | Estimate | Parameter (unit) | Estimate |
| *K*G (/week) | 0.0118 (23) | 54 (27) | Intercept | −3.49 (5.0) | λ (/ week) | 0.00596 (49) | λ (/week) | 0.0079 |
| *K*DRUG (/week/AUC) | 0.0050 (47) | 119 (61) | θPD | 1.12 (12) | α | 1.23 (6.9) | α | 1.15 |
| *K*sKIT (/week) | −0.00282 (89) | 243 (38) | θSLD (/mm) | 0.00105 (50) | β1 Tumor base (/mm) | 0.00237 (28) | β1 Tumor base (/mm) | −0.00172 |
| *KsVEGFR-3 (/week)* | −0.0371 (30) | - | θTime (/week) | 0.00707 (54) | β2 sVEGFR-3 | 3.77 (16) | β3 dBPREL | −1.29 |
| λ (/week) | 0.0217 (32) | - | - | - | λcens (/week) | 0.0017 (46) | β4 ANC (l/.109) | 4.76 |
| Residual error (%) | 12.5 (20) | - | - | - | αcens | 1.27 (6.6) | λcens (/week) | 0.0019 |
|  |  |  |  |  |  |  | αcens | 1.27 |

AUC = area under the concentration–time curve; CV = coefficient of variation; IIV = interindividual variability; *K*_G_ = tumor growth rate constant; *K*_DRUG_ = tumor size reduc- tion rate constant; *K*sKIT = tumor size reduction rate constant; *K*_sVEGFR-3_ = tumor size reduction rate constant; sKIT = soluble stem cell factor receptor; sVEGFR = soluble VEGF receptor; VEGF = vascular endothelial growth factor; λ = resistance appearance rate constant; θ_PD_ = parameter related to occurrence of disease progression; θSLD = parameter related to tumor size at dropout; θTime = parameter related to time since start of study; λ = scale factor in the Weibull probability density function; α = shape factor in Weibull probability density function; β1 Tumor base = parameter relating observed baseline tumor size to the hazard; β2 sVEGFR-3 = parameter relating sVEGFR-3 to the hazard; β_3_ ANC, parameter relating ANC(t) to the hazard; β_4_ dBPREL, parameter relating the relative change in dBP to hazard;

λ_cens_ = scale factor in the Weibull probability density function for censoring; α_cens_ = shape factor in Weibull probability density function for censoring.
