# Supplementary material for: Model-Based Biomarker Selection for Dose Individualization of Tyrosine-Kinase Inhibitors
Source: Front Pharmacol. 2020 Mar 12;11:316. doi: 10.3389/fphar.2020.00316 (PMC7080977; doi:10.3389/fphar.2020.00316)
Supplement: Supplementary file 2 [file Table_2.docx]

**Table S1.2** Parameter estimates for axitinib pharmacokinetic model (relative SEs, %) (Garrett et al) *

|  | Final estimate | %RSE | 95% CI |
| --- | --- | --- | --- |
| CL (l h−1) | 17.0 | 6.6 (8.2) | 14.9, 19.4 |
| Vc(l) | 45.3 | 6.4 (8.3) | 40.0, 51.3 |
| Weight effect on Vc | 0.758 | 14 (14) | 0.556, 0.960 |
| Q (l h−1) | 1.74 | 9.2 (16) | 1.45, 2.08 |
| Vp (l) | 45.9 | 25 (57) | 28.0, 75.3 |
| ka (h−1) | 0.523 | 7.6 (7.0) | 0.450, 0.607 |
| Form XLI* | −0.150 | 23 (23) | −0.219,−0.0814 |
| tlag(h) | 0.457 | – | – |
| ω2 CL | 0.272 | 14 (11) | 0.208, 0.355 |
| ω2 Vc | 0.0949 | 25 (18) | 0.0579, 0.155 |
| ω2 Q | 0.406 | 22 (27) | 0.266, 0.619 |
| ω2 Vp | 1.07 | 33 (44) | 0.566, 2.02 |
| ω2 ka | 0.506 | 13 (13) | 0.392, 0.654 |
| ω CL ω Vc | 0.141 | 22 (15) | 0.0812, 0.201 |
| ω Q ω Vp | 0.619 | 26 (35) | 0.300, 0.938 |
| RUV Oral, % | 50.9 | 2.7 (2.9) | 48.3, 53.7 |

CI = conﬁdence interval; CL = systemic clearance; F= absolute bioavailability; ka = ﬁrst-order rate of absorption; Q = peripheral clearance; RSE = relative standard error; tlag, absorption lag time; Vc = central volume of distribution; Vp = peripheral volume of distribution.

* In simulations the absolute bioavailability for the XLI formulation was fixed to 0.530 following the formula:

0.465 x 1.338 x 0.85; *bioavailability absolute x relative bioavailability fasted x relative bioavailability XLI*.

**Table S2.2** Parameter estimates for sVEGFR-3 biomarker model (relative SEs, %) (Schindler et al)

| Parameter | Typical value (RSE%) | IIV %CV (RSE%) |
| --- | --- | --- |
| Base (pg/mL) | 19,500 (6.5) | 49 (15) |
| MRT (days) | 5.76 (12) | — |
| Imax | 1 | — |
| AUC50 (ug·h/L) | 717c (8.6) | 45 (17) |
| γ | 1 | — |
| α (year-1) | — | — |
| RUV | 0.263 (6.5) | — |
| Common RUV^a^ | 0.0593 (26)e | — |

sVEGFR-3 = soluble vascular endothelial growth factor receptor 3; IIV = individual variability; CV = coefficient of variation; Base = baseline biomarker concentration; MRT = mean residence time; Imax = maximal inhibitory effect; AUC50 = axitinib area under the concentration-time curve giving half of the maximal effect; γ = Hill coefficient; α = slope of the disease progression; RUV = residual unexplained variability.
aCommon RUV for all four biomarkers.

**Table S3.2** Parameter estimates for tumor size, dropout, and survival model (relative SEs, %) (Schindler et al)

| Parameter | Estimate (RSE%) | IIV %CV (RSE%) |
| --- | --- | --- |
| Tumor size model | |  |
| KG (week-1) | 0.00361 (1.8) | 160 (20) |
| ksVEGFR-3 (week-1) | -0.174 (15) | - |
| γ (week-1) | 0.101 (18) | 72 (16) |
| RUV (%) | 10.5 (8.2) | 35 (21) |
| Dropout model | |  |
| σ_0_ | 26.11 (7.4) | - |
| σ_PD_ | 1.22 (22) | - |
| σ_SLD_ (mm-1) | 0.00282 (31) | - |
| σ_AUC_ (L·h-1·ug-1) | -0.00529 (18) | - |
| σ_Time_ (day-1) | 0.00371 (45) | - |
| Diastolic blood pressure model | | |
| dBP0 (mmHg) | 78.9 (1.4) | 6.7 (12) |
| Shape_dBP0_ | 25.42 (42) | - |
| MRT_dBP_ (days) | 4.92 (19) | - |
| Emax,dBP | 0.197 (14) | - |
| S0_,dBP_ (L·h-1·ug-1) | 0.00127 (50)a | - |
| RUV (mmHg) | 6.13 (7.0) | - |
| Overall survival model | |  |
| β_0_ | 7.09 (3.2) | - |
| γ | 0.298 (22) | - |
| β_SLD_ (mm21) | 0.0115 (17) | - |

KG = tumor growth rate constant; ksVEGFR-3 = tumor size reduction rate con- stant related to soluble vascular endothelial growth factor receptor 3 (sVEGFR-3) response, which is negative since sVEGFR-3rel(t) is negative (reduction from baseline); k = tumor resistance/regrowth appearance rate constant; RUV = residual unexplained variability; h0, intercept of the logistic regression model; hPD = coefficient for the effect of occurrence of progressive disease; hSLD = coefficient for the effect of sum of longest diameters (SLD) at the time of evaluation; hAUC = coefficient for the effect of axitinib daily area under the curve (AUCdaily); hTime = coefficient for the effect of time since start of study; dBP0 = baseline diastolic blood pressure; ShapedBP0 = shape parameter in the Box-Cox transformation of dBP0 random effects; MRTdBP = mean residence time of dBP response; Emax,dBP = maximum axitinib effect on diastolic blood pressure; S0,dBP = slope of the Emax model; b0 = scale parameter of the log-logistic baseline hazard model; γ = shape parameter of the log- logistic baseline hazard model; bSLD = coefficient for the effect of longitudinal SLD on the hazard.
